# Analysis of the influencing factors of the unsafe driving behaviors of online car-hailing drivers in china

**DOI:** 10.1371/journal.pone.0231175

**Published:** 2020-04-02

**Authors:** Yun Xiao

**Affiliations:** School of Urban Construction and Transportation, Hefei University, Hefei, Anhui, China; Tongii University, CHINA

## Abstract

Online car-hailing drivers are a special group between professional drivers and private car drivers. The paper built the unsafe driving behavior model of online car-hailing drivers based on the theory of planned behavior (TPB), explored the socio-psychological factors underlying drivers’ motivation for unsafe driving behavior and examined how these factors predict their behaviors. 239 online car-hailing drivers were surveyed with a self-reported questionnaire. Factors analysis proved the TPB questionnaire to be valid and reliable. Structural equation modeling showed that attitude to behavior (0.18), subjective norm(0.39) significantly influenced drivers' behavioral intention, perceived behavioral control (0.27) could both affected drivers' behavioral intention (0.27) and behavior(0.21),behavioral intention was the most direct and important predictor of behavior. This study provided a valuable contribution to designing more effective interventions to improve driving safety of online car-hailing drivers.

## Introduction

Online car-hailing refers to the business activities of building a service platform based on Internet technology and providing non-cruise-booking taxi services, which brings many benefits, such as reducing the use of private cars[1,2], easing traffic congestion, improving the environment[3,4]. As a new business model, online car-hailing first emerged in Europe and America, and has been increasing in popularity since entering China in 2012 [5]. In 2018, the traffic volume of online car-hailing reached 20 billion times, and the traffic volume of cruise taxi reached 35.2 billion times, the traffic volume of online car-hailing accounted for 36.2% of the total traffic volume in china [6].

With the rapid development of online car-hailing, the traffic accidents related to car-hailing are also growing rapidly. Taking Shenzhen city as an example, there are 3879 traffic accidents involving online car-hailing in 2017, the probability of traffic accidents involving online car-hailing is 7.15%, while the probability of traffic accidents involving taxi and private car is 1.78% and 0.28% respectively in the same period[7]. As a new mode of transportation between private cars and public transportation, there are few studies on safety of online car-hailing. Rayle et al. [8] argued that online car-hailing may offer a safer service than traditional taxi by reducing drunk driving. While Sun XJ [9] revealed online car-hailing drivers often pay attention to the APP client during driving to rush for passenger orders quickly, which may increase risk of traffic accidents. Some researchers [10, 11] hold that online car-hailing experience a higher risk than private vehicle, partly because of their much higher levels of exposure on the roads.

Driver’s factors account for a large proportion of transportation accidents [12, 13].Unsafe behaviors of drivers can be divided into three categories: errors, lapses and violations [14]. Researchers noted that driving behavior is influenced easily by distracted driving, leading to traffic accidents [15]. Drivers with certain characteristics are more prone to unsafe on the road, and some types of situations cause drivers to become angry when driving [16,17]. To study the relationship between driving behavior characteristics and personality traits, the complex reactions, speed estimation and personality traits of the subjects were studied, and inter individual differences related to driving behavior were noted [18, 19, 20].

The theory of planned behavior (TPB) provides a theoretical framework to study the relationship between personality and behavior. On the basis of the theory of planned behavior (TPB), Zhao et al. [21] proposed a perception-norm-execution (PNE) driving simulator-based training model. A Previous research aimed to predict the drivers' safe-driving behaviors based on TPB and habit, and come to conclusion that habits showed to be a stronger predictor of safe-driving behaviors than attitude and perceived behavioral control [22]. Chen et al. [23] investigates the efficacy of TPB in predicting self-reported engagement behavior in a number of distraction tasks through the assessment of attitudes, perceived behavioral control, descriptive norms, and injunctive norms.

As a special group, online car-hailing drivers experience high road traffic exposure, but most of them don’t have professional training, which leads to a higher risk of traffic accidents than other drivers. The aim of this study was to use a sample population of online car-hailing drivers to investigate unsafe behavior in China. This investigation involved the revision of the TPB, the verification of its reliability and validity, the exploration of differences in unsafe behavior among different driver personality types, and the examination of unsafe behaviors in relation to demographic factors. All of the factors related to unsafe behavior were considered to determine whether it was possible to predict unsafe behavior significantly among online car-hailing drivers.

## Methods

### Ethical statement

The study was approved by the Institutional Review Committee of the School of Urban Construction and Transportation at Hefei University. Before implementing the study, our research plan was discussed by several experts. They believed that the questionnaire would not cause any mental injury to the participants, nor would it have any negative social impact or affect the participant. As a consequence, they agreed that the research plan was scientifically sound and feasible, and comply with laws and regulations in China.

In addition, at the beginning of the questionnaire, there is an option: "are you willing and agree to complete this survey?”, all the participants voluntarily decided whether to continue. All information related to the participants is strictly confidential. We obtained informed consent from the participants, informing them that the results of the survey would be used only for academic research and that would not have any negative impact on them. Follow-up research can only be carried out with their permission. The investigation was conducted in a voluntary and anonymous manner.

### Research hypothesis

The TPB is based on the theory of reasoned action (TRA), with the addition of a new construct—perceived behavioral control (PBC). As a new behavior theory research model which includes the five elements of attitude to behavior (AB), subjective norm (SN), perceived behavioral control (PBC), behavioral intention (BI), and behavior (B). AB refers to a person's positive or negative feelings about the behavior, and SN refers to the social pressure that individuals feel when they take a certain behavior. PBC refers to the reflection of personal past experience and expected obstacles, when individuals think that the more resources and opportunities they have, the fewer obstacles they can expect, then the stronger their behavioral control will be. BI refers to the determination of the subjective probability an individual’s willingness to adopt a specific behavior. B refers to the behavior in which an individual actually takes action.

**Fig 1:**
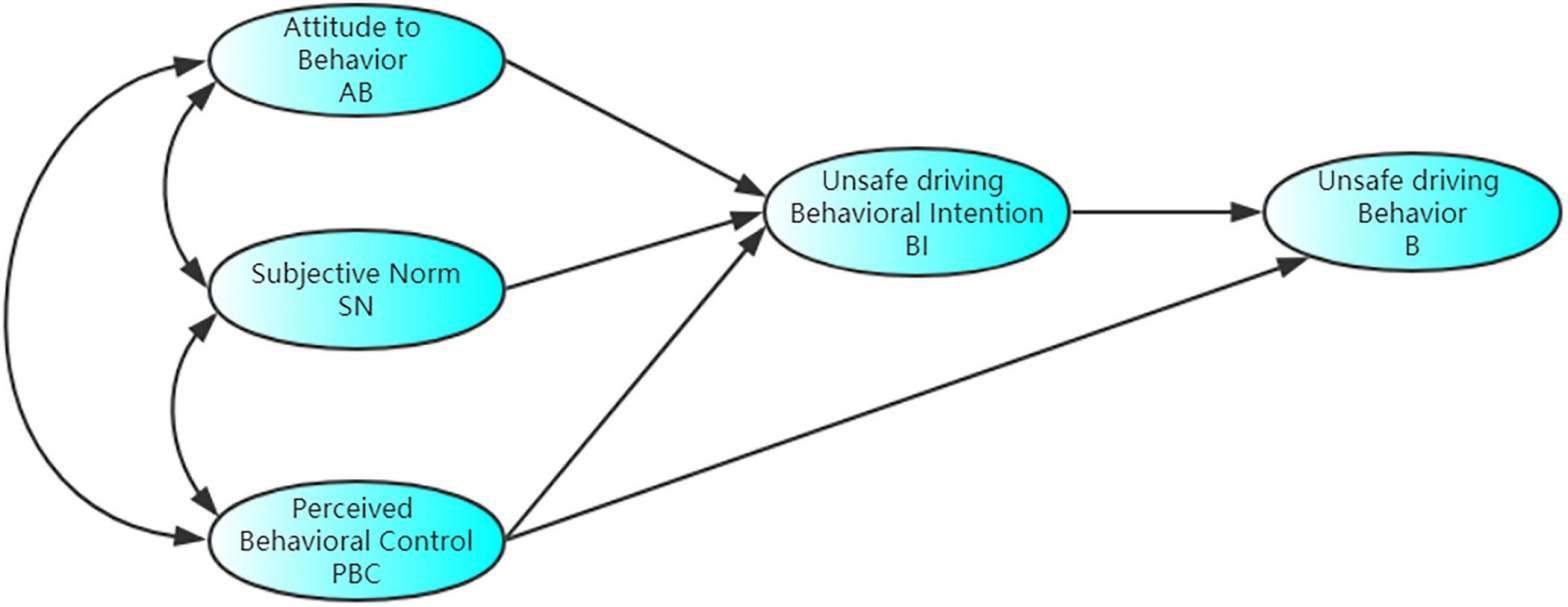
TPB model for unsafe driving behavior.

Base on the TPB and review of related studies [24, 25, 26], we formulated some hypotheses and developed a conceptual model, as is shown in Fig 1.

H1: The behavioral intention toward unsafe behaviors of online car-hailing is predicted by attitude, subjective norms and perceived behavioral control.

H2:Self-reported unsafe behaviors of online car-hailing is predicted by behavioral intention.

H3: Self-reported unsafe behaviors of online car-hailing is predicted by perceived behavioral control.

H3a: There is an association between perceived behavioral control and self-reported unsafe behaviors through behavioral intention.

### Analysis of unsafe driving behavior of online car-hailing drivers

**Fig 2:**
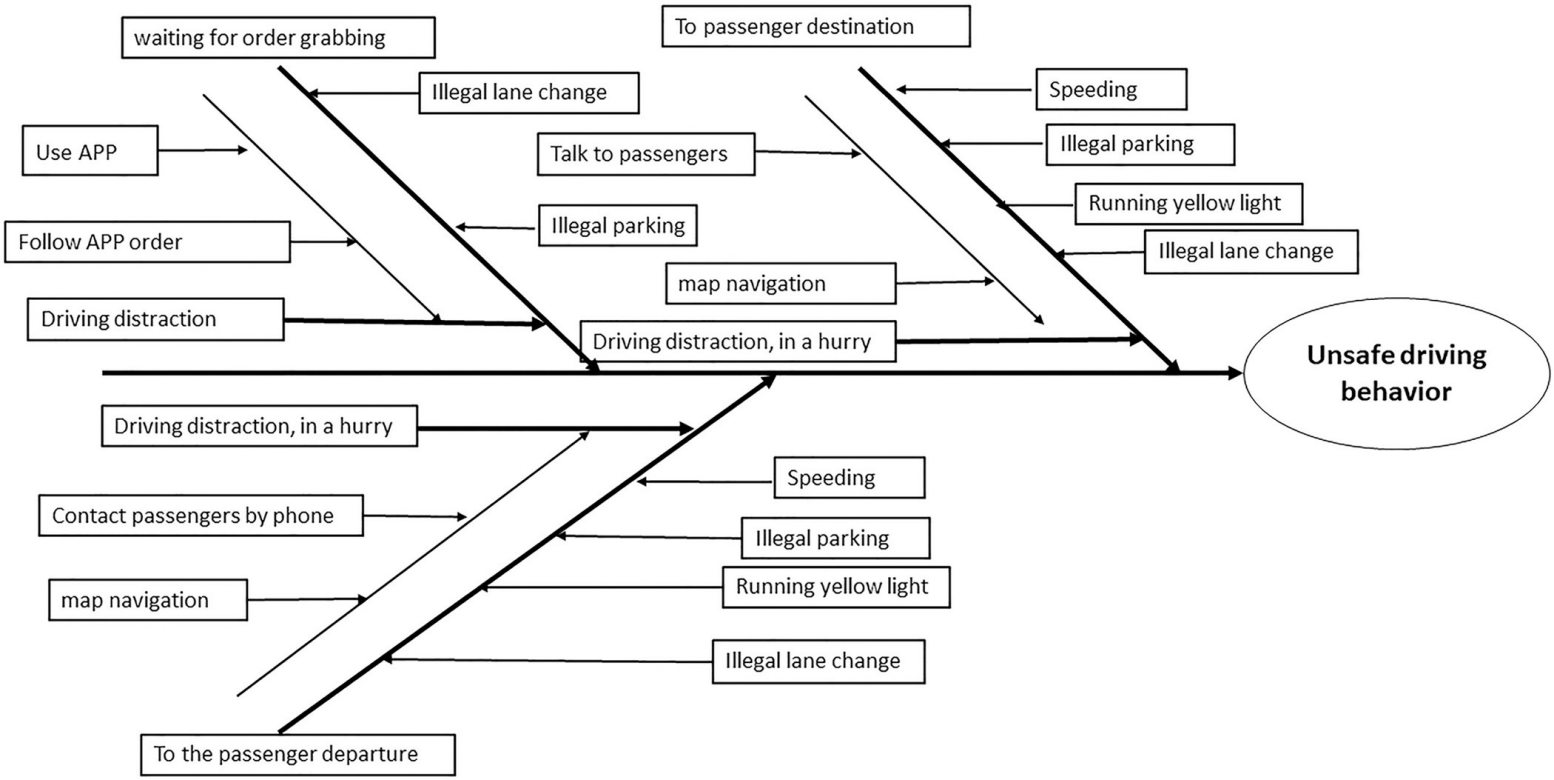
Fishbone diagram of unsafe behavior.

The fishbone diagram was used in order to analyze unsafe driving behavior of online car-hailing drivers, fishbone diagram can clearly show causes of problems and have an overall grasp of problem solving. The operation process of online car-hailing was divided into three stages: waiting for order (receiving dispatching), going to the passenger's departure place and going to the passenger's destination, as shown in Fig 2.

During the operation, online car-hailing drivers are often distracted, such as using APP to grab orders, answering and calling passengers. Distracted behaviors have a significant negative effect on traffic safety. When distracted, drivers' access to road information is reduced, and they are more prone to speeding, illegal lane change, running yellow light and other behaviors [27, 28].

### Questionnaire survey

#### Participants and procedure

The 301 online car-hailing drivers were asked to report their driving behaviors with the cooperation of the Hefei Transit Authority, who from different companies in Hefei city, china.

A total of 239 questionnaires were complete and valid, representing 79.4% of the total questionnaires administered. All results were anonymously self-reported. The study removes invalid questionnaires in two ways, one is to fill in incomplete or abnormal questionnaires, such as fill in the same data for all columns; the other is that drivers work less than 2 hours a day, who are not representative.

#### Questionnaire design

The questionnaire design includes demographic information and TPB variables. The demographic information mainly including age, gender, working hours and driving information.

The TPB model mainly consists of five constructs: attitude to behavior, subjective norms, perceived behavioral control, behavioral intention and unsafe behavior. Each construct has 3–4 questions to reduce survey errors. All questions was measured on a 5-point scale, ranging from 1 = ‘‘strongly disagree” to 5 = ‘‘strongly agree”.

Attitude to behavior mainly to test the belief and evaluation of online car-hailing drivers of the violation. Subjective norm mainly to test beliefs and motivation to comply with unsafe driving behaviors. Perceived behavioral control mainly to test the control beliefs and perceived power of drivers. Behavioral intention mainly indicates the driver's intention to engage in unsafe behavior. Unsafe behavior indicate how often drivers violate the traffic rules in the past month.

Five constructs and 16 items were adopted based on the proposed model and related studies on traffic violations [29], which are shown in Table 1.

**Table 1:** Main questionnaire indicators in this study.

Constructs	Items	Statements
Attitude to behavior(AB)	AB1	I feel guilty when driving unsafely
	AB2	I feel disgusted when others are driving unsafely
	AB3	occasionally driving unsafely is unavoidable
Subjective Norm(SN)	SN1	my family is opposed to my driving unsafely
	SN2	I care about my family's views
	SN3	My friends(company) are very opposed to my driving unsafely
	SN4	I care about my friends(company)’ views
Perceived Behavioral Control(PBC)	PBC1	It will affect my handling of the vehicle when driving unsafely
	PBC2	It will prone to traffic accidents when driving unsafely
	PBC3	I can match the high challenge of driving unsafely
Behavioral intention(BI)	BI1	It is likely that I intend to violate traffic rules if I feel my car is capable of doing safely
	BI2	It is likely that I intend to violate traffic rules when I am in hurry
	BI3	It is likely that I intend to violate traffic rules when passenger ask to do so
Unsafe behavior(B)	B1	How often do you parking illegally in the past month
	B2	How often do you illegal lane change in the past month
	B3	How often do you speed in the past month
	B4	How often do you run yellow light in the past month

## Results

### Demographics and descriptive variables

Participants’ demographic information is described in Table 2. Participants included 211 (88.3%) male and 28 (11.7%) female drivers. Descriptive statistics showed that most of the Participants (47.3%) were between the ages of 30 and 40. Most of Participants (54.0%) had between 1 and 3 years’ experience of online car-hailing driving.

**Table 2:** Driver’s demographic information (N = 239).

Items	Freq.	percent	Items	Freq.	percent
**Age**			**Gender**		
**<30**	46	19.3%	**Male**	211	88.3%
**30–39**	113	47.3%	**Female**	28	11.7%
**40–49**	45	18.8%	**Work experience(Year)**		
**> = 50**	35	14.6%	**<1**	65	27.2%
**Work hours per day(hour)**			**1–3**	129	54.0%
**<4**	51	21.3%	**>3**	45	18.8
**4–8**	130	54.4%	**Penalty points in the last year**		
**>8**	58	24.3%	**0**	91	38.1%
**accidents in the past three years**			**1–3**	47	19.7%
**0**	51	21.3%	**4–6**	62	25.9%
**1**	47	19.7%	**6–9**	20	8.4%
**2**	74	30.9%	**>9**	19	8.0%
**3**	47	19.7%			
**>3**	20	8.4%			

Table 2 also summarizes the average number of traffic accidents in which the participants were involved during the past three years and total penalty points received for violations in the past year. In summary, only 21.3% of participants have no traffic accidents in the past three years, and 38.1% of participants didn’t received penalty points.

### Reliability and validity test of the questionnaire

A reliability analysis was conducted, and the Cronbach Alpha value of internal consistency of the total scale was 0.91. Furthermore, the internal consistencies of the four dimensions were 0.87 (attitude to behavior), 0.85 (subject norm), 0.87 (perceived behavioral control) and 0.88 (behavioral intention). The Cronbach Alpha value was greater than 0.7 for the all dimensions, thus indicating acceptable reliability, these results demonstrated that the TPB model exhibited good internal reliability.

An additional validity analysis of the TPB model was performed by using SPSS 23.0. The results are shown in Table 3. The KMO coefficient is 0.878, which is greater than 0.50, and the Sig value is 0.00, which is less than 0.05. Thus, factor analysis can be performed.

**Table 3:** KMO and Bartlett's test.

**Sufficient sampling of KMO metrics**		0.878
**Bartlett's spherical test**	**Approximate chi-square**	2652.29
	**df**	136
	**Sig**	0.000

### Analysis of age differences

An ANOVA revealed significant differences among the four groups on total unsafe behavior (F = 11.10, p < 0.01) and on the four items: illegal parking (F = 10.95, p < 0.01), illegal lane change (F = 5.74, p < 0.01), speeding (F = 6.29, p < 0.01), running yellow light (F = 10.53, p < 0.01). The results (Table 4) indicated that drivers under 30 are more likely to have unsafe driving behaviors (M = 2.71, SD = 0.79), and drivers older than 50 are less likely to experience unsafe driving behavior (M = 1.94, SD = 0.64).

**Table 4:** Analysis of age differences.

Items	Age				F value	p
	< = 30	31–40	41–50	> 50		
Illegal parking	3.53±1.14	3.40±1.33	2.64±1.32	2.51±0.85	10.95	0.00**
Illegal lane change	2.65±1.25	2.51±0.91	2.07±0.70	1.94±1.06	5.74	0.00**
Speeding	2.48±1.06	2.51±1.00	2.02±0.70	1.82±0.91	6.29	0.00**
Running yellow light	2.17±0.77	2.12±0.79	1.51±0.53	1.46±0.67	10.53	0.00**
Total	2.71±0.79	2.64±0.74	2.06±0.55	1.94±0.64	11.10	0.00**

*p<0.05,

**p<0.01.

### Analysis of gender differences

There are significant differences in gender on total unsafe behavior (F = 4.92, p < 0.05) and on the four items: illegal parking (F = 8.19, p < 0.05), illegal lane change (F = 6.28, p < 0.05), speeding (F = 6.29, p < 0.01), running yellow light (F = 3.92, p < 0.05). The results (Table 5) indicated that male are more likely to have unsafe driving behaviors (M = 2.41, SD = 0.66), and female are less likely to experience unsafe driving behavior (M = 2.05, SD = 0.49).

**Table 5:** Analysis of gender differences.

Items	Gender		F value	p
	Male	Female		
Illegal parking	3.24±1.34	2.57±1.29	8.19	0.01*
Illegal lane change	2.43±1.04	1.93±0.59	6.28	0.01*
Speeding	2.34±1.05	2.10±0.61	6.29	0.00**
Running yellow light	1.96±0.84	1.61±0.47	3.92	0.04*
Total	2.41±0.66	2.05±0.49	4.92	0.03*

*p<0.05,

**p<0.01.

### Analysis of work experience

There are significant differences among the three groups on total unsafe behavior (F = 3.55, p < 0.05) and on the two items: illegal parking (F = 4.26, p < 0.05), speeding (F = 3.42, p < 0.03).while there are no significant differences on the other items: illegal lane change and running yellow light. The results (Table 6) indicated that unsafe driving behavior will decrease as working experience increase.

**Table 6:** Analysis of work experience.

Items	work experience(year)			F value	p
	<1	1–3	>3		
Illegal parking	3.48±1.47	3.08±1.21	2.86±1.3	4.26	0.02*
Illegal lane change	2.57±1.19	2.37±0.88	2.2±0.71	2.02	0.14
Speeding	2.46±0.97	2.36±1.04	1.98±0.84	3.42	0.03*
Running yellow light	2.01±0.86	1.95±0.82	1.69±0.62	1.96	0.14
Total	2.63±0.80	2.44±0.76	2.18±0.65	3.55	0.03*

*p<0.05.

**p<0.01.

### Analysis of work hours

There are no significant differences among the three groups on total unsafe behavior. While an ANOVA revealed significant differences on speeding item (F = 3.29, p < 0.05), and the results (Table 7) indicated that drivers who work more than 8 hours per day have higher unsafe driving behaviors (M = 2.63, SD = 0.80).

**Table 7:** Analysis of work hours per day.

Items	Work hours			F value	p
	< 4 hours	4–8 hours	> 8 hours		
Illegal parking	3.18±1.31	3.04±1.36	3.36±1.29	1.52	0.22
Illegal lane change	2.35±0.95	2.38±0.92	2.45±0.99	0.14	0.88
Speeding	2.33±0.95	2.18±0.94	2.59±1.12	3.29	0.04*
Running yellow light	1.90±0.77	1.85±0.74	2.10±0.97	1.67	0.19
Total	2.44±0.73	2.36±0.75	2.63±0.80	1.78	0.17

*p<0.05.

**p<0.01.

### Analysis of penalty points in the last year

There are significant differences among the five groups on total unsafe behavior (F = 6.78, p < 0.01) and on the four items: illegal parking (F = 4.37, p < 0.01), illegal lane change (F = 4.36, p < 0.01), speeding (F = 5.10, p < 0.01), running yellow light (F = 7.56, p < 0.01). The results (Table 8) indicated that drivers who received above 9 points are more likely to have unsafe driving behaviors (M = 3.34, SD = 0.81), and drivers with no penalty are less likely to experience unsafe driving behavior (M = 2.29, SD = 0.63).

**Table 8:** Analysis of penalty points in the last year.

Items	Penalty points in the past year					F value	p
	No penalty	1–3 points	4–6 points	7–9 points	Above 9 points		
Illegal parking	2.93±1.15	3.17±1.14	3.11±1.35	3.30±1.59	4.10±1.54	4.37	0.00**
Illegal lane change	2.23±0.89	2.27±0.68	2.40±0.83	2.65±1.29	3.15±1.14	4.36	0.00**
Speeding	2.16±0.80	2.19±1.07	2.27±1.12	2.55±0.89	3.21±0.73	5.10	0.00**
Running yellow light	1.83±0.71	1.78±0.56	1.77±0.70	2.15±0.87	2.89±1.10	7.56	0.00**
Total	2.29±0.63	2.36±0.63	2.39±0.77	2.66±0.88	3.34±0.81	6.78	0.00**

*p<0.05.

**p<0.01.

### Analysis of number of traffic accidents in the past three year

There are significant differences among the five groups on total unsafe behavior (F = 4.12, p < 0.01) and on the three items: illegal parking (F = 3.78, p < 0.01) and illegal lane change (F = 4.17, p < 0.01) and speeding (F = 2.86, p < 0.05), while there are no significant differences on running yellow light. The results (Table 9) indicated that drivers who have more than 3 accidents are more likely to have unsafe driving behaviors (M = 2.35, SD = 1.08), and drivers with 0 accident are less likely to experience unsafe driving behavior (M = 2.26, SD = 0.46) or 1(M = 2.15, SD = 0.72).

**Table 9:** Analysis of accident in the past three years.

Items	Accidents in the last three years					F value	p
	0	1	2	3	>3		
Illegal parking	2.96±1.20	2.79±1.34	3.16±1.45	3.44±1.12	3.75±1.04	3.78	0.00**
Illegal lane change	2.10±0.57	2.11±0.92	2.53±0.97	2.66±0.93	2.70±1.27	4.17	0.00**
Speeding	2.22±0.77	2.00±0.87	2.32±0.99	2.53±1.25	2.75±1.04	2.86	0.02*
Running yellow light	1.78±0.49	1.72±0.68	1.96±0.86	2.02±0.98	2.35±1.08	2.23	0.07
Total	2.26±0.47	2.15±0.72	2.49±0.82	2.66±0.82	2.88±0.87	4.12	0.00**

*p<0.05.

**p<0.01.

### Results of the structural model

According to the TPB model, drivers' attitude to behavior, subjective norm, and perceived behavioral control can predict driver behavior. The SEM is used for modeling, the advantage of SEM is that it can clearly analyze the role of the individual indicators overall and in the relationships among the individual indicators. Different icons in the above structure path diagram have different meanings: rectangles represent observed variables or factors, ellipses represent latent variables or factors, one-way arrows represent single effects or effects, and two-way arc arrows express correlations. The model is constructed by using AMOS 23.0.

According to the data from the questionnaire, the values of various observations and some latent variables in the model are obtained to establish a structural path diagram of the unsafe driving behavior.

**Fig 3:**
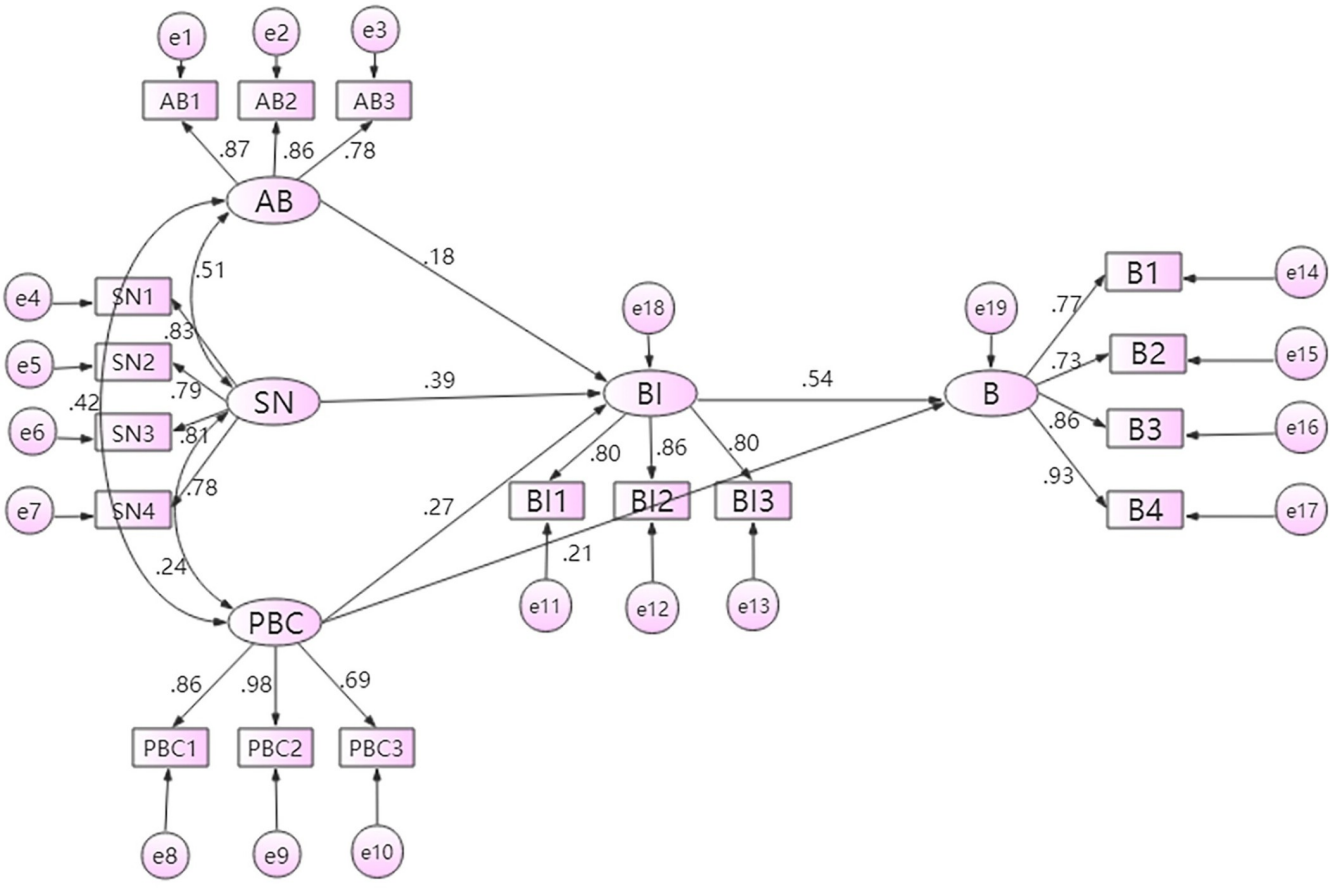
Structural model of the unsafe driving behavior of online car-hailing drivers.

The structural model fit indices (NFI, CFI, GFI, AGFI, PGFI, PNFI, RMSEA, and PCLOSE) satisfy all acceptable criteria listed in Table 10, indicating a sufficient basis for path analysis. All hypotheses proposed in this study were tested, all paths represent significant standardized regression weights (Std.R.W.). The structural model is shown in Fig 3.

**Table 10:** The structural model fit indices.

Indices	P value for RMSEA	Goodness of fit index	Adjusted GFI	Root mean square error of approximation	Normed fit index	Comparative fit index
**Abbreviation**	PCLOSE	*GFI*	*AGFI*	RMSEA	NFI	CFI
**Recommended criteria**	P>0.05	>0.90	>0.80	<0.05	>0.90	>0.90
**Value**	0.680	0.924	0.895	0.046	0.939	0.979
**Degree of fit**	Good	Good	Good	Good	Good	Good

## Discussion

The paper presented a comprehensive analysis on unsafe behavior of online car-hailing drivers with TPB model. Previous studies [21, 22, 23] showed the TPB model could successfully predict and explain driving behaviors, and diving behaviors is related with factors such as distracted [29], speeding [13], anger driving [16]. The paper confirmed previous studies, and found that AB, SN, PBC could predict online car-hailing drivers’ behavioral intention toward unsafe behaviors, such as illegal parking, illegal lane change, speeding and running yellow light. Moreover, drivers’ unsafe behavior could be predicted by behavioral intention and PBC, hypothesis H1-3 is supported.

Our study revealed that online car-hailing drivers behaved differently from other drivers, the most frequent violation of online car–hailing drivers is illegal parking (M = 3.16, SD = 1.17), while the most frequent violations of other drivers are speeding, scrambling et al [14, 17, 30]. Unlike cruising taxis, online car-hailing drivers are often necessary to wait for passengers during the operation, which is an important reason why the online car-hailing drivers are more prone to illegal parking. Private car drivers experienced less time on the road, which means the risk of violations is also lower.

The effects of behavioral intention on unsafe driving behavior is 0.54, which is the most direct and strongest predictor of behaviors, and indicated the role of rational decision-making in online car-hailing drivers’ unsafe behaviors. Previous studies come to similar conclusions [22, 23, 26]. Therefor changing online car-hailing drivers’ behavioral intention is the key factor to improve the safety of online car-hailing.

AB affect drivers’ intention toward unsafe behaviors (Std.R.W. = 0.18). The result show many drivers don’t pay enough attention to violation and hold that occasionally driving unsafely is unavoidable. Related studies believe that Strengthening rule education can promote drivers to drive safely [26].Moreover online car-hailing drivers are likely to risk driving unsafely because it will bring them meaningful benefits, such as saving time, getting more orders, or earning more money.

SN (Std.R.W. = 0.39) significantly affect drivers’ intention toward unsafe behaviors. Most of their family and friends are opposed unsafe behaviors, and the opinion of drivers’ family and friends have significant impact on their unsafe behaviors. So an effective driving safety improvement measure must play the role of family, friends, so that online car-hailing drivers realize that their unsafe behaviors are not recognized by the society. Moreover traffic safety education should not only for drivers, but also for their family and friends as intervention groups. In this way, drivers may get a positive impact from their family and good friends.

PBC affects drivers’ behavioral intention (Std.R.W. = 0.27) and behavior (Std.R.W. = 0.21), indicating that driver's behavioral intentions for unsafe behaviors depend to a large extent on their ability to control themselves. PBC can bypass behavioral intention and directly affect behavior, improving drivers’ PBC are effective ways to reduce unsafe behaviors.

## Conclusions

Online car-hailing drivers are a special group between professional drivers and private car drivers. This study uses the TPB and a structural equation model to study the mechanism of the unsafe driving behaviors of online car-hailing drivers. The influence relationship between external and internal dependent variables is described. Overall, the study found evidence supporting that behavioral intention and PBC are both significantly related to self-reported unsafe driving behavior of online car-hailing drivers. Behavioral intention is the most direct and important predictor, which is also significantly related to attitude to behavior, subjective norms, and perceived behavioral control. Perceived behavioral control can predict self-reported unsafe driving behavior both directly and indirectly. These findings could provide a valuable contribution to designing more effective interventions to improve driving safety of online car-hailing drivers.

### Limitations of the present study

This study has certain limitations and must be considered when interpreting the results. Because this study is based on drivers’ self-reported data, there is a bias in social expectations. Although participants were guaranteed complete confidentiality and anonymity, and were geographically separated from researchers even during the survey, the usual shortcomings of self-reported questionnaires were inevitable. Therefore, a more comprehensive survey will enhance the accuracies of results.

## Supporting information

S1 File The data of this study. (XLSX)
